# 

**Published:** 1985-04

**Authors:** 


APRIL 1985

VOLUME 100 (ii)

NUMBER 374

